# Supplementary figures and images for: Differential susceptibility of male and female germ cells to glucocorticoid-mediated signaling
Source: eLife. 2024 Jan 16;12:RP90164. doi: 10.7554/eLife.90164 (PMC10945581; doi:10.7554/eLife.90164)

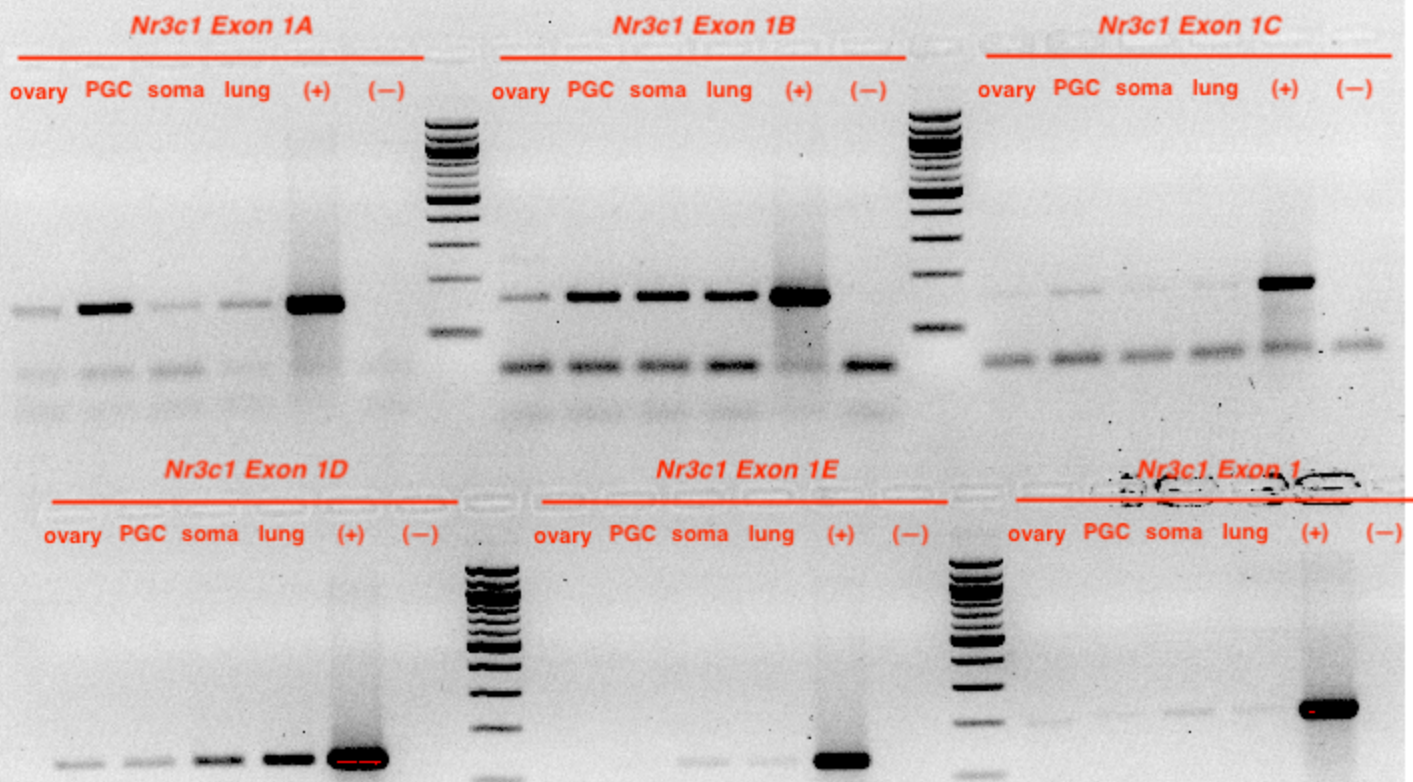

Supplement: Figure 1—source data 1. [file elife-90164-fig1-data1.pdf]

***Nr3c1* Exon 1G**

ovary PGC soma lung (+) (-)

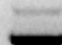

***Nr3c1* Exon 2-3**

ovary PGC soma lung (+) (-)

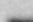

***Ddx4***

ovary PGC soma lung (+) (-)

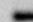

Supplement: Figure 1—source data 2. [file elife-90164-fig1-data2.pdf]

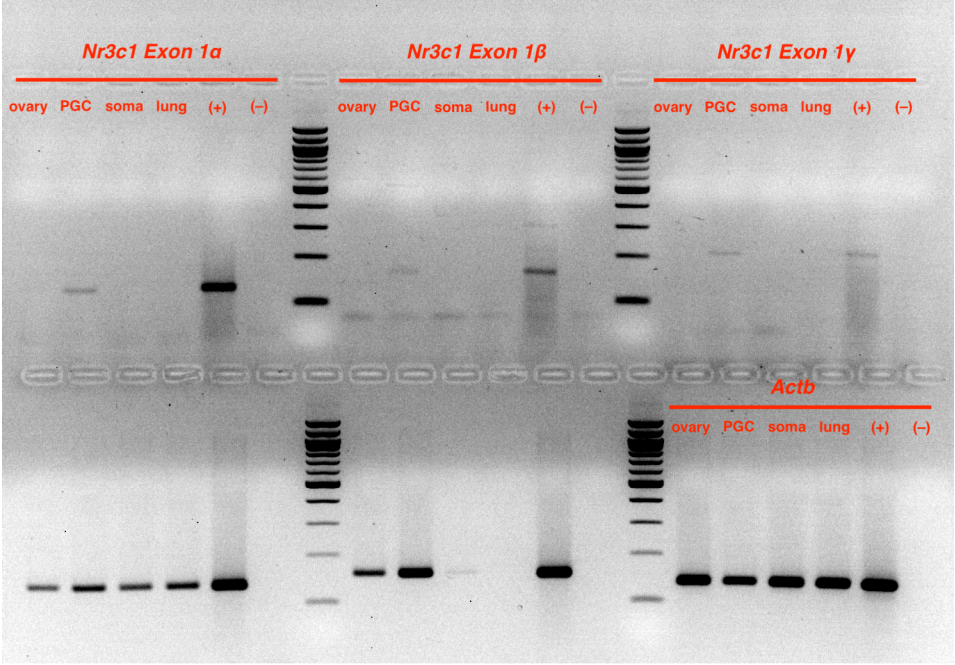

Supplement: Figure 1—source data 3. [file elife-90164-fig1-data3.pdf]

**E**

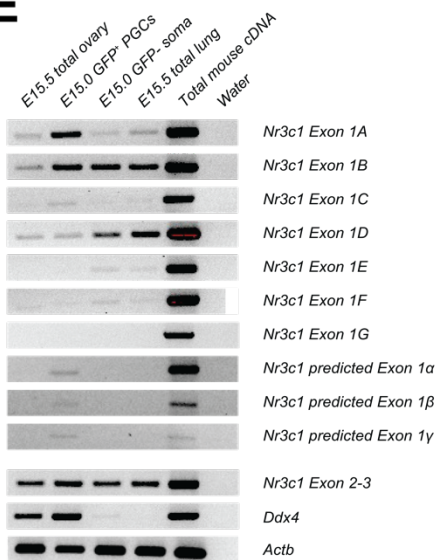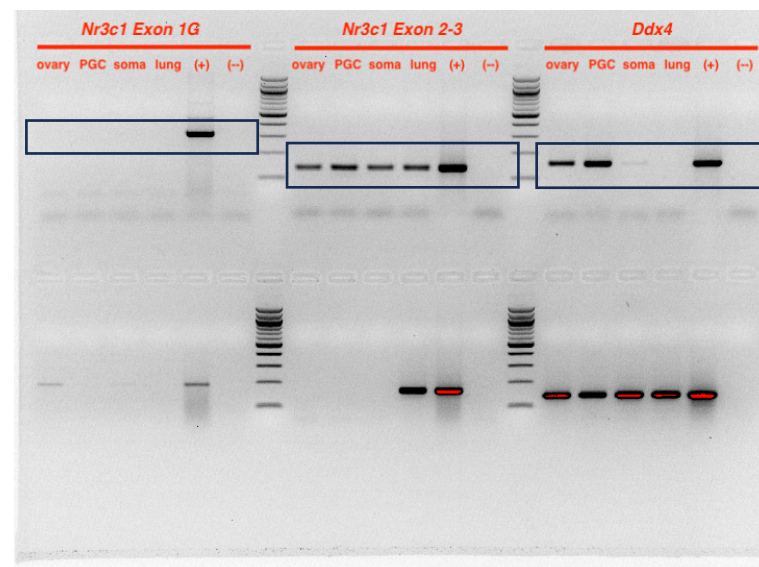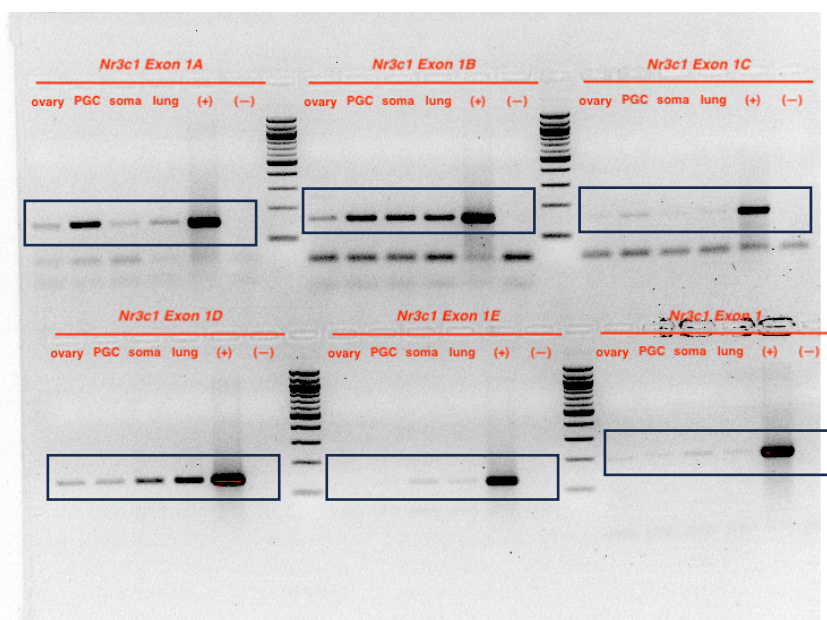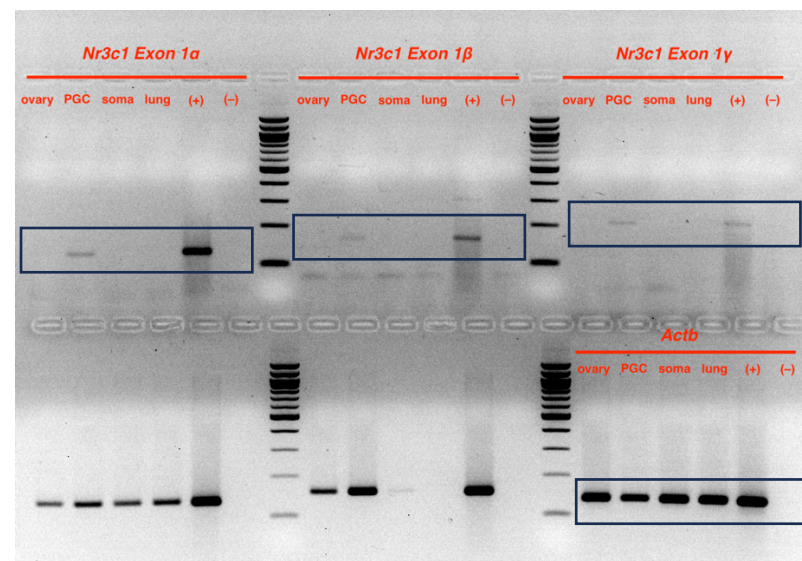

Supplement: Figure 1—source data 4. [file elife-90164-fig1-data4.pdf]

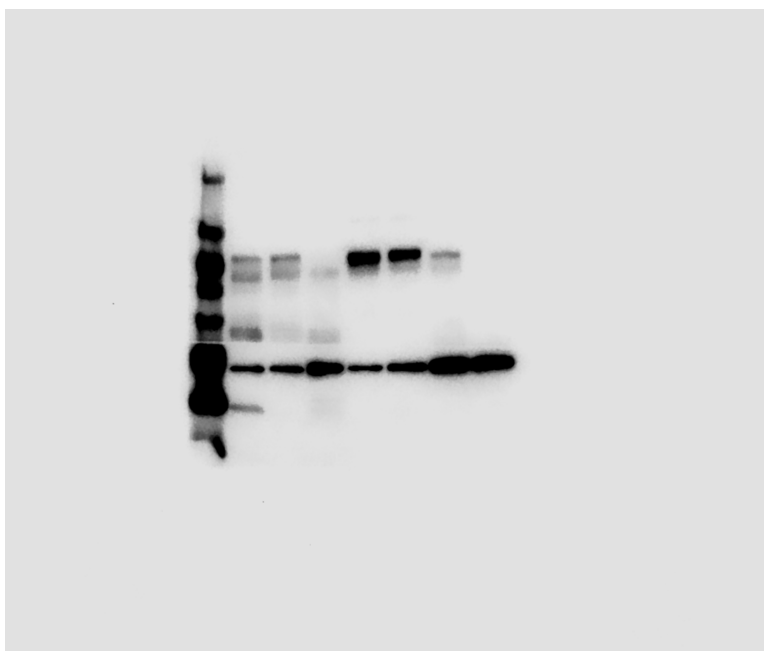

Supplement: Figure 2—source data 1. [file elife-90164-fig2-data1.pdf]

**B**

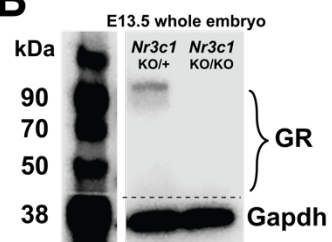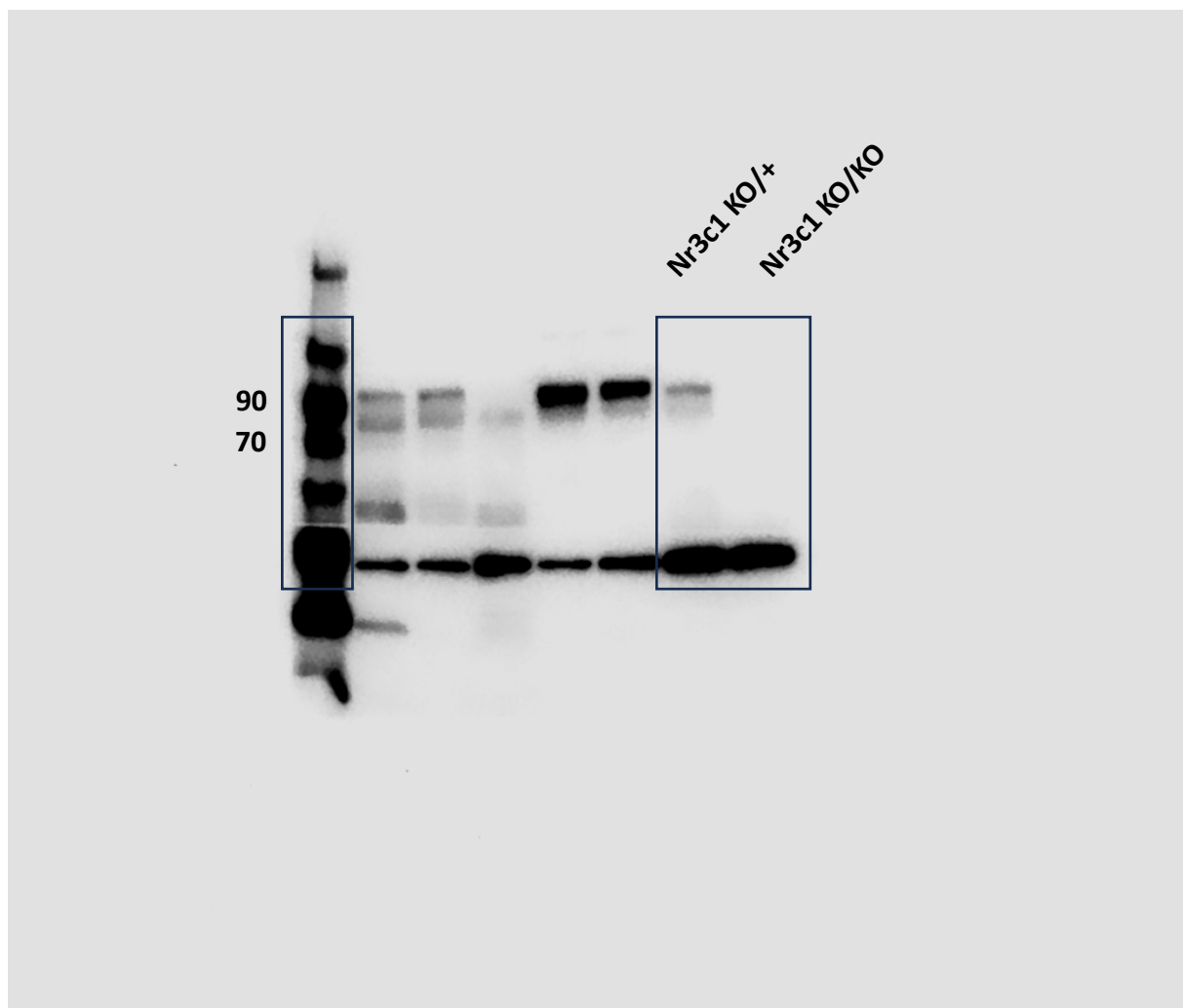

Supplement: Figure 2—source data 2. [file elife-90164-fig2-data2.pdf]

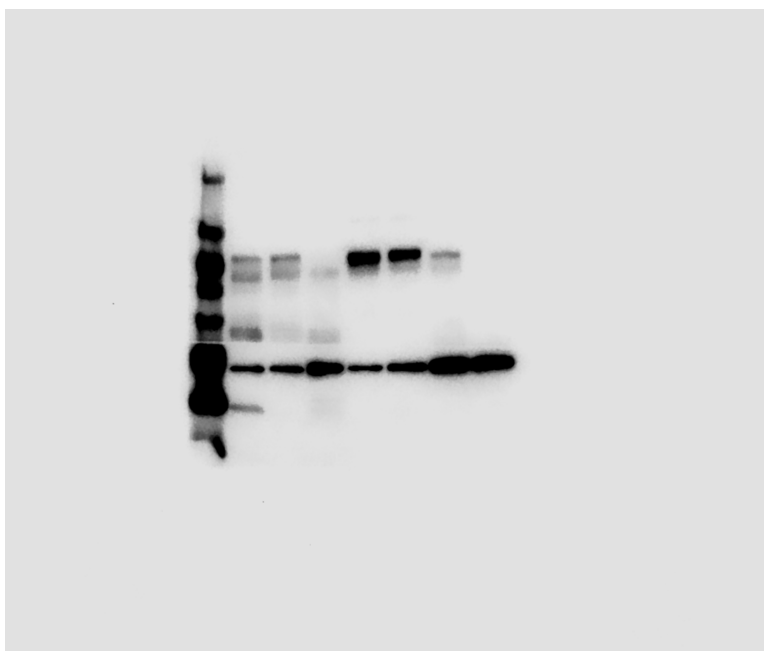

Supplement: Figure 3—source data 1. [file elife-90164-fig3-data1.pdf]

**C**

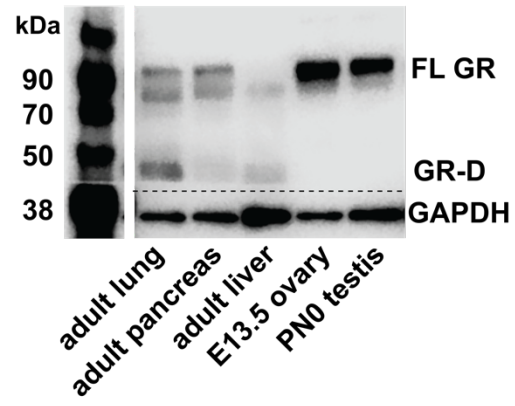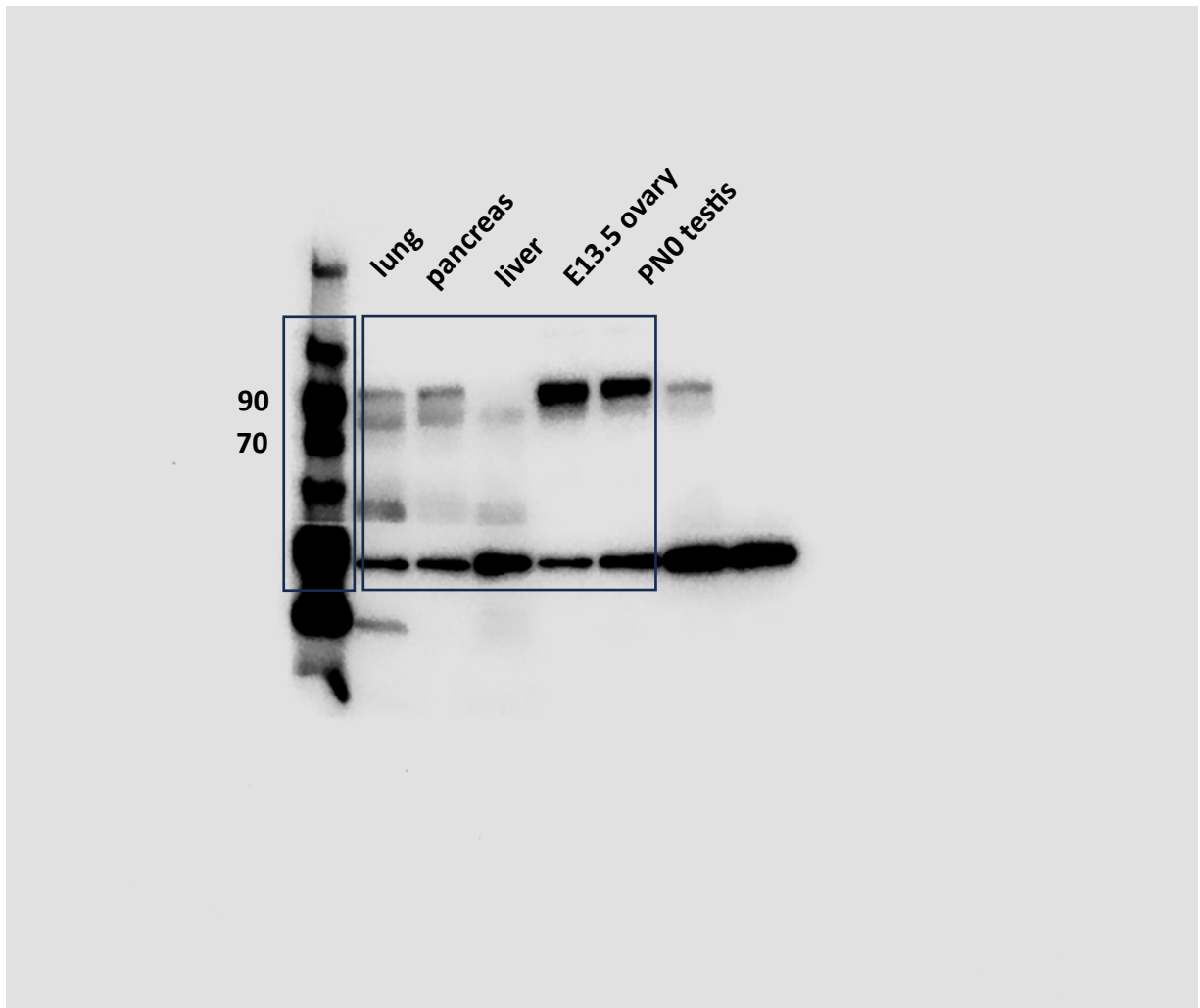

Supplement: Figure 3—source data 2. [file elife-90164-fig3-data2.pdf]
